# Hemodynamic and Morphological Parameters of Ruptured Mirror Posterior Communicating Artery Aneurysms

**DOI:** 10.3389/fneur.2021.653589

**Published:** 2021-09-27

**Authors:** Jinlong Yuan, Chenlei Huang, Zhenbao Li, Xiaochun Jiang, Xintong Zhao, Degang Wu, Nianshen Lai, Jiaqiang Liu, Bingbing Zhang, Feiyun Qin, Dayong Xia, Xinggen Fang

**Affiliations:** ^1^Department of Neurosurgery, The First Affiliated Hospital of Wannan Medical College (Yijishan Hospital of Wannan Medical College), Wuhu, China; ^2^Department of Clinical Laboratory, The First Affiliated Hospital of Wannan Medical College (Yijishan Hospital of Wannan Medical College), Wuhu, China

**Keywords:** computational fluid dynamic, hemodynamics, morphological, mirror aneurysms, posterior communicating artery

## Abstract

**Objective:** Morphological and hemodynamic parameters might predict rupture of intracranial aneurysms (IAs). A practical model for the study is patients with ruptured mirror IAs in which one is ruptured and the other is unruptured. Although there have been analyses of the morphology and hemodynamics of ruptured mirror posterior communicating artery aneurysms (PComAAs), the sample sizes in these studies were small and only considered hemodynamics or morphological characters. Therefore, this study aimed to investigate the morphological and hemodynamic parameters associated with ruptured mirror PComAAs.

**Methods:** We considered 72 patients with ruptured mirror PComAAs using computational fluid dynamics (CFDs). Ruptured mirror PComAAs were divided into ruptured and unruptured groups. Fourteen morphological and eight hemodynamic parameters were calculated and compared. Significant parameters were analyzed by the multivariate logistic regression to identify independent risk factors. Receiver operating characteristic (ROC) analysis was performed, and the area under the ROC curve (AUC) was calculated for all independent risk factors to determine the predictability and identify the optimal threshold.

**Results:** Four hemodynamic and three morphological parameters were significantly different between ruptured and unruptured groups: normalized wall shear stress (NWSS), mean WSS, low wall shear WSS area (LSA%), size, aspect ratio (AR), size ratio (SR), and inflow angle (IA). Multivariate logistic regression analysis showed that AR, SR, NWSS, mean WSS, and LSA% were all independent factors significantly associated with PComAAs rupture. The ROC analysis for independent risk factors indicated that AR (0.751), NWSS (0.755), mean WSS (0.69), and LSA (0.778) had merely acceptable AUC values. Only SR (0.803) had a high acceptable AUC value. The threshold value of SR was 1.96.

**Conclusions:** SR (>1.96) was the most significant parameter associated with IA rupture, whereas AR, NWSS, mean WSS, and LSA independently characterized the status of IA rupture.

## Introduction

With the development and popularization of non-invasive cerebrovascular technology, unruptured intracranial aneurysms are more often detected in about 3–7% of the population (1, 2). The rupture of intracranial aneurysms (IAs) is the principal cause of subarachnoid hemorrhage (SAH), leading to tremendous mortality and disability (3). Because these aneurysms have a relatively low risk of rupture, and both endovascular and clipping treatment may cause complications during surgery, it is crucial to balance the risks of rupture and complications (4). Consequently, it is advisable to predict the risk of aneurysm rupture and identify high-risk IAs for further treatment.

Although the mechanism of IA rupture is exceptionally complex and is not entirely clear, hemodynamic, and morphological parameters are thought to be vital factors in the pathogenesis, progression, and rupture of IAs (5–7). By using computational fluid dynamics (CFD) in the medical field, it is possible to quantify hemodynamics. Several studies on morphological or hemodynamic risk factors for intracranial IA rupture returned confusing results (8–11). Cebral et al. found that high WSS (wall shear stress) was associated with IA rupture (10). However, Xiang et al. considered that low WSS and high OSI (oscillatory shear index) were related to the rupture of aneurysm (5). This may have been caused by differences in individual patients and aneurysm locations. Ruptured mirror aneurysms are defined as the situation in which one is ruptured, and the other is not. This is an ideal model to identify parameters associated with rupture (12–14). Posterior communicating artery aneurysms (PComAAs) account for about 15–25% of all IAs and are one of the most likely to rupture (15). Accordingly, we performed the research on the morphologic and hemodynamic analysis of 72 patients with ruptured mirror PComAAs. This is the most significant number of mirror PComAAs studied to identify risk factors associated with IA rupture to the best of our knowledge.

## Materials and Methods

The Research Ethics Committee of Wannan Medical College approved the study, which was conducted according to the Declaration of Helsinki. All subjects were selected from the Department of Neurosurgery at the First Affiliated Hospital of Wannan Medical College, Wuhu City, China. The subjects and their families provided informed written consent.

### Patient Selection

**Figure 1:**
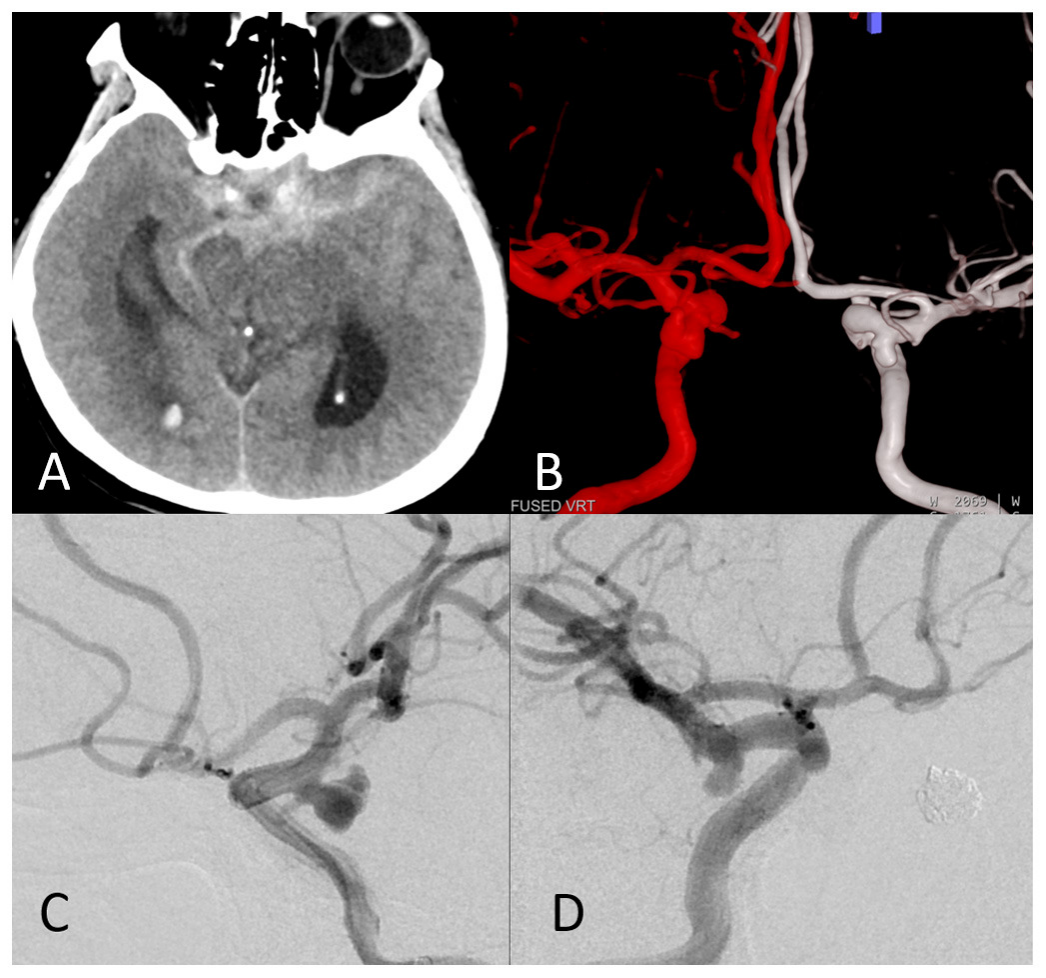
The images of a representative ruptured mirror posterior communicating artery aneurysm (PComAA). A 67-year-old woman presented with a severe headache. **(A)** Brain computed tomography (CT) scan showed subarachnoid hemorrhage (SAH) more on the left side. **(B)** Dual fusion of three-dimensional-digital subtraction angiography (3D-DSA) revealed a mirror PComAA. The left side of the mirror PComAA was more irregular than the right side. **(C)** The left internal carotid artery angiography revealed an irregular posterior communicating artery aneurysm that was a ruptured aneurysm. **(D)** The right internal carotid artery angiography showed a regular posterior communicating artery aneurysm.

We retrospectively reviewed our database from January 2013 to December 2019. A total of 78 consecutive patients with ruptured mirror PComAAs were diagnosed and treated at our hospital. The SAH was confirmed using brain computed tomography (CT), and all patients with ruptured mirror PComAAs were diagnosed by computed tomography angiography or digital subtraction angiography (DSA) after admission. The inclusion criteria were as follows: the paired sidewall aneurysms located at the PComA with one ruptured and the other one unruptured, and the quality of DSA images was adequate for CFD analysis. Exclusion criteria were as follows: (1) no SAH history, or the rupture site was hardly recognized; (2) fusiform or dissecting, mycotic aneurysms and true posterior communicating aneurysms; (3) three-dimensional (3D) DSA imaging was too poor for CFD analysis. Ultimately, 72 patients with 144 PComAAs were divided into two groups (ruptured and unruptured aneurysms) (Figure 1). The clinical features of 72 patients with ruptured mirror PComAAs are displayed in Table 1.

**Table 1:** Clinical features of 72 patients with ruptured mirror PComAAs.

**Variable**	**Value**
Total	72
Age	58.18 ± 11.22
Female (%)	57 (79.17%)
**Rupture site**	
Rt	33 (45.83%)
Lt	39 (54.17%)
**Hunt and Hess grades**	
I	5 (6.94%)
II	47 (65.28%)
III	14 (19.45%)
IV	6 (8.33%)
Family SAH (%)	7 (9.72%)
Family history of aneurysms (%)	11 (15.28%)
Hypertension (%)	29 (40.28%)
Diabetes (%)	10 (13.89%)
Drinking (%)	8 (11.11%)
Smoking (%)	6 (8.33%)
Hyperlipidemia (%)	23 (31.94%)
Atherosclerosis (%)	15 (20.83%)
CI (%)	5 (6.94%)
CHD (%)	12 (16.67%)

*Rt, right; Lt, left; CI, Cerebral infarction; CHD, Coronary heart disease; SAH, subarachnoid hemorrhage*.

### Imaging

A total of 144 three-dimensional reconstruction models were acquired using DSA (Siemens, Germany, Artis zee Floor VC14). One second after injection of contrast medium (15 ml in total), the images were collected by 360° rotation with 15 frames per second for 8 seconds. With the Syngo X Workplace workstation, the collected 266 frames were rebuilt into unique patient 3D models and then output in stereolithography (STL) format. The result of transcranial Doppler of parent vessel velocity waveform was collected from a normal subject. We used MATLAB 14.0 (MathWorks, Natick, MA, US) to calculate the flow velocity waveform curve of the whole cardiac cycle for the subsequent CFD analysis.

### Establishment of Patient-Specific IAs Models

The unique patient models were trimmed and smoothed in GEOMAGIC STUDIO 12.0 (Geomagic, Morrisville, North Carolina). The trimmed models were inputted to ICEM CFD 14.0 (ANSYS, Canonsburg, Pennsylvania, US) to generate finite grids for subsequent CFD analysis. The maximum element size was set to 0.3 mm. In this study, each model was divided into 120000–1600000 grids. After that, 14.0 (ANSYS Inc., USA) software set the blood properties, vessel boundary conditions, and calculation steps. Each cardiac cycle of 0.8 s was divided into 800 steps, each step of 0.001s. The simulation process assumed that the blood flow was incompressible and laminar, governed by the N-S equation. Viscosity coefficient (μ) and blood density (ρ) were set at 0.00345 pa·s and 1050 kg /m^3^, respectively. The boundary condition of the vessel wall was set as a rigid wall and no sliding boundary. The blood flow condition at the vascular entrance was set as flow velocity waveform curve obtained by previous transcranial Doppler. The outlet condition was set as no stress, and the static pressure was zero. Three cardiac cycles were calculated to obtain more stable simulation data, and the last cycle was exported.

### Morphological Parameter Calculation

We measured 13 previously defined morphological parameters (16). All the morphological parameters were divided into 2D and 3D groups. The 10 2D parameters, included size, size ratio (SR), aspect ratio (AR), bottleneck factor (BNF), depth/width ratio (HWR), inflow angle (InA), vessel angle (VA), aneurysm inclination angle (AIA), irregular shape, and bifurcation. These were investigated using 3D-DSA workstation. In addition to 2D morphological parameters, the other three 3D variables, ellipticity index (EI), undulation index (UI), and non-sphericity index (NSI), were calculated by the method of Lv et al. (8). The fetal-type posterior cerebral artery (FPCA) was defined as a PCA that originates entirely from the internal carotid artery or has a slight connection with the basilar artery. To acquire the accurate results morphological parameters, measurements, and calculations were performed by two skilled neurosurgeons (WD, LN).

### Hemodynamic Parameter Calculation

A total of eight hemodynamic factors were divided into qualitative and quantitative factors. We measured five quantitative hemodynamic parameters, including NWSS, mean WSS, LSA%, OSI, and RRT. RRT, OSI, and mean WSS were directly computed by CFD. (1) Mean WSS, that is, the time-averaged WSS, represented merged WSS at each point over the entire cardiac cycle. The threshold was 0–20 pa (2). The OSI, defined as measuring the directional change in WSS during the cardiac cycle, was a non-dimensional parameter. The threshold was 0–0.2. (3) RRT reflected the residence time of blood flow near the wall. The threshold was 0–1.0 s. The three hemodynamic parameters were computed using the methods of Xiang et al. (5). (4) NWSS meant that the WSS of the aneurysm was normalized by WSS of the parent artery (5). The LSA was described by the area of aneurysm wall exposed to the WSS below 10% of the mean WSS of the parent artery. The LSA% was that LSA standardized by the dome area (5, 17).

The qualitative hemodynamic parameters, such as flow stability, inflow concentration, and impingement zone, were described by Cebral et al. and calculated by two neurosurgeons with experience with CFD analysis (10). (6) The flow stability was the stable blood flow pattern persevered during the cardiac cycle. By contrast, the unstable flow meant that the flow pattern changed during the cardiac cycle (18). (7) Compared with the aneurysm neck, the concentrated inflow jet was thin or narrow in the main flow direction (8). The small impingement size was the area of the impingement region <50% area of the aneurysm. On the contrary, it was called a large impingement size (18).

### Statistical Analysis

We used Shapiro–Wilkes W test to calculate whether the parameters were normal distribution for quantitative variables. If the parameters were normal distribution, data were described as means ± SDs and used two-tailed independent Student *t*-tests to analyze the differences. If the parameters were abnormally distributed, data were described as deviation medians(interquartile range) and Mann–Whitney *U* test to analyze the differences. For categorical variables, the χ^2^ test was used to compare the differences. The differences were statistically significant if *p*-values were less than 0.05. Significant parameters were calculated by multivariate logistic regression analysis to identify independent parameters. The ROC analysis was used to acquire cut-off values on the independent parameters. All the analyses and calculations were used SPSS Statistics version 20.0 (SPSS, Inc., Chicago, Illinois, US) and MedCalc version 15.0.0 (MedCalc Software bvba, Ostend, Belgium).

## Results

### Clinical Characteristics

There were 72 patients with ruptured mirror PComAAs, including 57 females and 12 males. These were divided into ruptured and unruptured groups. Patient age ranged from 34 to 83 years, with a mean age of 58.18 years (deviation 11.22 years). In terms of health habits, there were six (8.33%) smokers and eight (11.11%) alcohol drinkers. For family history, there were seven (9.72%) patients with a family history of SAH and 15 (15.28%) patients with a family history of aneurysms. With respect to comorbidities, there were 29 patients (40.28%) with hypertension, 23 (31.94%) with hyperlipidemia, 10 (13.89%) with diabetes, 15 (20.83%) with atherosclerosis, five patients (6.94%) with cerebral infarction history, and 12 (16.67%) with coronary heart disease. According to the Hunt-Hess scale, there were 5 patients (6.94%) in grade I, 47 (65.28%) in grade II, 14 (19.45%) in grade III, and 6 (8.33%) in grade IV. The clinical characteristics of patients with 72 ruptured mirror PComAAs are displayed in Table 1.

### Morphologic Factors

As shown in Table 2, the size, AR, SR, and IA in the ruptured group were significantly larger than those in the unruptured group (*p* < 0.001, *p* < 0.001, *p* < 0.001, and *p* = 0.029, respectively, Table 2). Nevertheless, the BNF, HWR, VA, AIA, EI, UI, FPCA, and NSI showed no significant differences between groups (Table 2). The BNF, HWR, VA, EI, UI, FPCA, and NSI in the ruptured group were higher than those in the unruptured group. However, the difference in AIA was the opposite. The ruptured group was more likely to have irregularly shaped and bifurcated aneurysms than the unruptured group; however, the difference was not statistically significant (Table 2).

**Table 2:** Result of univariate statistical analysis for morphological parameters.

**Paramaters**	**Ruptured (*n* = 72)**	**Unruptured (*n* = 72)**	***p***
Size(mm)	5.70 ± 2.45	4.86 ± 2.23	<0.001
AR	1.58 ± 0.55	1.13 ± 0.37	<0.001
SR	2.33 ± 0.74	1.54 ± 0.47	<0.001
BNF	1.49 ± 0.57	1.35 ± 0.43	0.091
HWR	1.18 ± 0.38	1.09 ± 0.28	0.089
Irregular shape [*n* (%)]	40 (55.56%)	29 (40.28%)	0.095
Bifurcation [*n* (%)]	43 (58.9%)	34 (48.6%)	0.18
FPCA [*n* (%)]	21 (29.2%)	17 (23.6%)	0.449
InA	119.95 ± 25.16	110.83 ± 21.83	0.029
VA	43.16 ± 18.31	38.20 ± 12.85	0.078
AIA	71.65 ± 22.69	75.91 ± 11.56	0.138
EI	0.18 ± 0.07	0.16 ± 0.06	0.073
UI	0.16 ± 0.08	0.14 ± 0.06	0.064
NSI	0.21 ± 0.07	0.19 ± 0.05	0.085

*AR, aspect ratio; SR, size ratio; BNF, bottleneck factor; HWR, height/width ratio; InA, inflow angle*.

*VA, vessel angle; AIA, aneurysm inclination angle*.

*EI, ellipticity index; UI, undulation index; NSI, non-sphericity index*.

*PcomA, posterior communicating artery*.

*FPCA, fetal-type posterior cerebral artery*.

### Hemodynamic Factors

**Figure 2:**
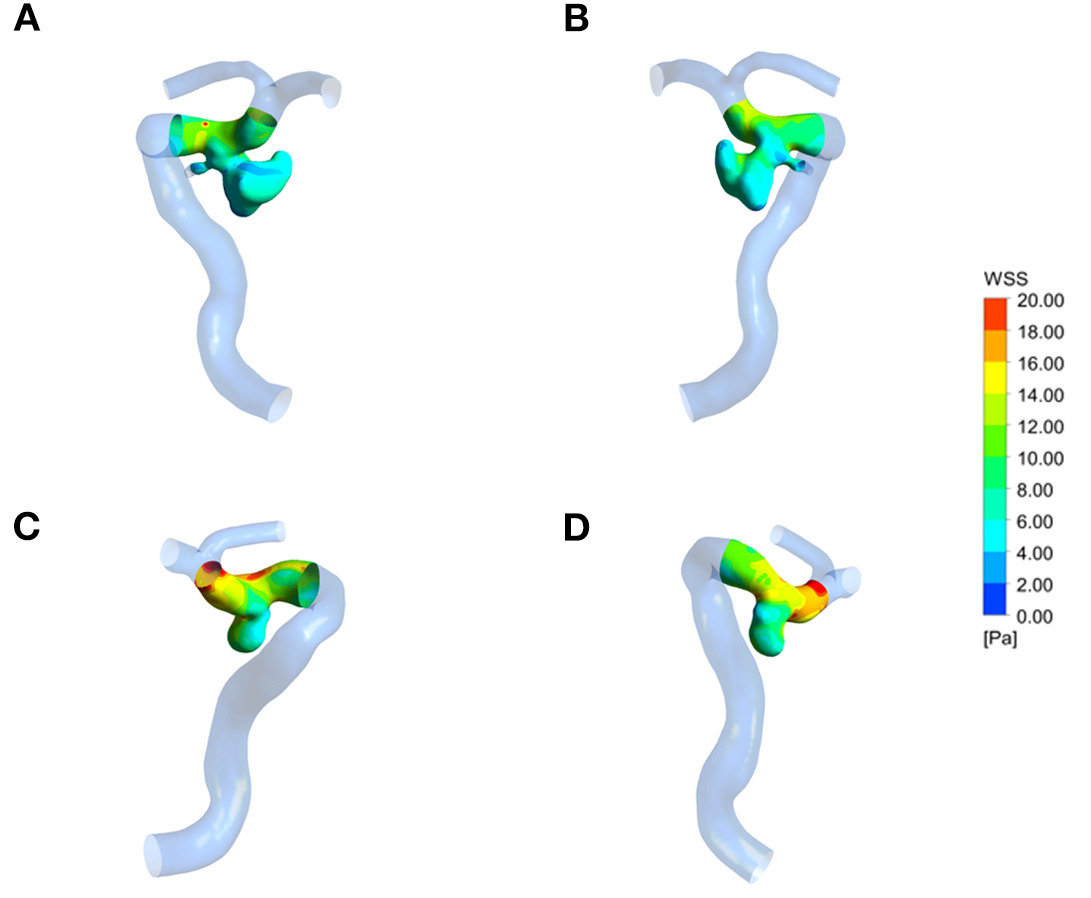
Wall shear stress (WSS) distribution of a representative ruptured mirror PComAA. **(A,B)** Two sides of WSS distribution of the ruptured left PComAA. **(C,D)** Two sides of WSS distribution of unruptured right PComAA. The WSS of ruptured PComAA was significantly lower than that of unruptured PComAA.

A total of 144 patient-specific models were reconstructed. The distribution of WSS in a ruptured mirror PComAAs is shown in Figure 2. Compared with the unruptured group, the ruptured group had significantly lower NWSS and mean WSS for quantitative hemodynamic parameters. Conversely, the LSA% in the ruptured group was significantly higher than that in the unruptured group. Although the results showed that the ruptured group usually had higher OSI and RRT than the ruptured group, the differences were not statistically significant (Table 3).

**Table 3:** Result of univariate statistical analysis for hemodynamic parameters.

**Paramaters**	**Ruptured (*n* = 72)**	**Unruptured (*n* = 72)**	***p***
Flow stability			0.132
Stable (%)	34 (47.22%)	44 (61.11%)	
Unstable (%)	38 (52.78%)	28 (38.89%)	
**Inflow concentration**			
Concentrated (%)	39 (54.17%)	32 (44.44%)	0.317
Diffused (%)	33 (45.58%)	40 (55.56%)	
Impingement size			0.066
Small (%)	43 (59.72%)	31 (43.06%)	
Large (%)	29 (40.28%)	41 (56.94%)	
NWSS	0.98 ± 0.41	1.49 ± 0.61	<0.001
Mean WSS(Pa)	8.20 ± 3.36	11.56 ± 5.28	<0.001
LSA(%)	9.80 ± 7.37	2.89 ± 2.73	<0.001
OSI	0.025 ± 0.015	0.023 ± 0.010	0.452
RRT	0.36 ± 0.26	0.30 ± 0.18	0.066

*WSS, Wall shear stress; NWSS, Normalized WSS; LSA, low WSS area; OSI, oscillatory shear index; RRT, relative residence time*.

Compared with the unruptured group, the difference between the two groups was not statistically significant for qualitative hemodynamic parameters, although ruptured aneurysms were more likely to have unstable flow patterns, concentrated inflow, and small impact size compared with the unruptured group (Table 3).

### Multivariate Logistic Regression and Receiver Operating Characteristics Analysis

To identify independent parameters predicting risk of rupture, multivariate logistic regression analysis was performed on significant morphological parameters (size, AR, SR, and IA) and hemodynamic parameters (NWSS, mean WSS, LSA%). As shown in Table 4, two morphological parameters (AR and SR) and three hemodynamic parameters (NWSS, mean WSS, and LSA) were independent predictors of IAs. The model showed that AR, SR, and LSA increased the risk of IAs rupture (Table 4). NWSS and mean WSS were inversely correlated with IAs rupture (Table 4).

**Table 4:** The result of multiple logistic regression analysis.

**Paramaters**	***P***	**OR**	**95%CI**
Size(mm)	0.397	1.145	(0.837, 1.156)
AR	0.007	9.032	(1.811, 45.040)
SR	0.002	5.853	(1.905, 17.982)
IA	0.207	1.019	(1.905, 17.982)
NWSS	<0.001	0.068	(0.017, 0.268)
Mean WSS(Pa)	0.005	0.740	(0.600, 0.913)
LSA (%)	0.001	1.437	(1.152, 1.792)

*p < 0.05 was considered statistically significant*.

**Figure 3:**
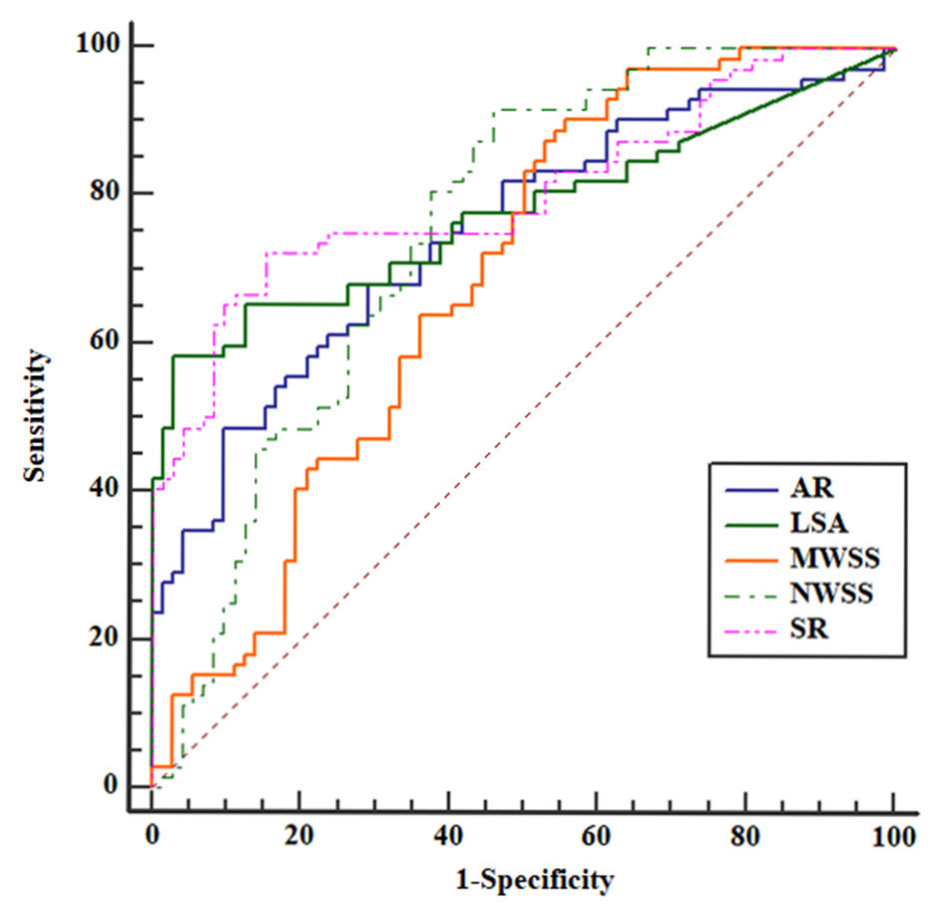
Receiver operating characteristic (ROC) curves for all the independent risk factors. The area under the curve (AUC) areas of size ratio (SR), aspect ratio (AR), LSA, mean WSS, and normalized wall shear stress (NWSS) were 0.803, 0.751, 0.778, 0.686, and 0.755, respectively. The SR had the largest AUC area.

To identify optimal thresholds for IA rupture, ROC analyses were performed for independent risk factors (Figure 3). AR (0.751), NWSS (0.755), mean WSS, and LSA (0.778) had merely acceptable AUC values. Only SR (0.803) had a high acceptable AUC value. The threshold value of SR was 1.96.

## Discussion

Morphological and hemodynamic parameters are associated with IA rupture. Controlling these parameters may help us identify the rupture risk characteristics. Ruptured mirror PComAAs with one ruptured and the other unruptured, at almost the same posterior communicating artery on each side in the same patient, may provide the ideal internal control. First, because mirror PComAAs with one ruptured and the other unruptured were from the same patient, the clinical parameters might be counterbalanced. Second, mirror PComAAs were situated on the posterior communicating artery on each side; therefore, this can rule out the influence of location associated with IAs. Third, in order to improve the accuracy of CFD, personalized boundary conditions were necessary. However, as a retrospective study, it was challenging to obtain personalized boundary conditions. A study using mirror PComAAs with one ruptured and the other unruptured may give more accurate results.

Although several recent studies with mirror PComAAs attempted to explain the relationship between hemodynamic or morphological and IA rupture, they all had some limitations (19–21) (Table 5). First, their sample size was small. This might lead to statistical bias and make the results inaccurate. Second, most previous studies were limited only to hemodynamics or morphology parameters. Third, mirror aneurysms are rare, comprising ~5% of all IAs, while ruptured mirror aneurysms located in the same location are rarer (14). Although previous studies selected mirror aneurysms located in different locations to avoid statistical bias, the accuracy of their results might be weakened by the location. We considered 14 morphological parameters and eight hemodynamic parameters with 72 ruptured mirror PComAAs models to identify rupture risk characteristics of IAs in the present study. SR was the most significant parameter associated with IA rupture, whereas AR, NWSS, mean WSS, and LSA independently characterized the status of IA rupture.

**Table 5:** Previous studies on hemodynamic or morphological analysis of mirror PComAAs.

**Study**	**Sample size**	**Hemodynamic analysis**	**Morphological analysis**	**Conclusion**
Xu et al. (19)	8	Yes	Yes	Lower WSS, higher LSA%, and higher AR.
Jiang et al. (20)	14	No	Yes	Higher size, AR, SR, and BNF. Bleb formation.
Wang et al. (21)	68	No	Yes	Higher AR and SR
Present	72	Yes	Yes	SR was the most significance.

*WSS, Wall shear stress; LSA, low WSS area; AR, aspect ratio; SR, size ratio; BNF, bottleneck factor; HWR, height/width ratio*.

Dhar et al. defined SR as the maximum height (size) divided by the average diameter of the parent arteries (22). A great majority of morphological parameters are concentrated in IA size. However, SR took into account not only the size of IAs but also the parent vessel diameter (location). It may provide promising prediction results. At the beginning of several previous studies, SR was a promising morphological parameter (23–25). In the subsequent studies with multiple IAs model, one ruptured and the others unruptured, higher SR was independently associated with rupture status (26–28). Although these studies showed that SR might be related to rupture risk, several potentially ignored problems might cast doubt on their results. At the beginning of the studies, location and individual variables, associated with rupture risk of IAs, were neglected. Although in the subsequent multiple IAs studies, avoiding the influence of individual characters, the location was also not considered. Second, the sample sizes of these studies were small.

Several morphological studies with mirror aneurysms, including location and individual variables, also provided confusing results. Xu et al. used eight mirror PComAAs and showed that SR had no relationship with aneurysm rupture (19). Fan, Tian, and Xu et al. used mirror aneurysms and reported that SR in ruptured IAs was higher than in unruptured IAs according to univariate analysis. However, SR was not an independent risk factor (13, 29, 30). Other studies on SR with mirror aneurysm models showed opposite results (20, 21, 31). Despite these studies took into account the influence of location and individual characteristics, they still ignored the hemodynamic factors and small sample sizes.

In the present study, we investigated hemodynamic and morphological parameters associated with rupture risk of IA rupture using 72 ruptured mirror PComAAs. We also found that SR was the most significant parameter associated with IA rupture. Tremmel et al. reported that, as SR increased, flow patterns became more complex and unstable and the LSA increased (32). Furthermore, the single aneurysmal vortex splits into multiple vortices, and LSA increases dramatically when the value of SR is greater than two. We also found that the threshold value of SR was 1.96, higher than reported in previous studies. This finding will need to be confirmed in future studies.

According to Ujiie et al. AR was defined as the ratio of maximum vertical height to average neck diameter. Most studies found that higher AR was an independent factor associated with aneurysm rupture. As AR increased, the height became great, or the neck became smaller, making the flow pattern complex and the circulation inside the aneurysm slow. These factors could trigger inflammatory changes in the aneurysm wall and then increase the risk of rupture (33, 34). Xu et al. studied morphological parameters in 48 ruptured mirror middle cerebral artery aneurysms and found that AR and anterior dome projection were independent rupture parameters (30). Wang et al. also reported that AR and SR were better predictors of rupture risk of IAs by using 68 patients with mirror PComAAs (21). These results agreed with those of the present study. In previous studies, the threshold value of AR ranged from 0.98 to 1.6, and the AR value of our study is 1.32, which was exactly in this range.

The WSS played an essential role in IAs occurrence, progression, and rupture. WSS is the frictional force of viscous blood, transformed into the biological signals. The signals regulate gene expressions and the cellular functions in vessel walls via mechanoreceptors on endothelial cells. For the hemodynamic studies using CFD for mirror aneurysms, higher LSA was related to rupture risk of IAs. Lu et al. showed that the LSA and OSI in ruptured aneurysms were higher in unruptured aneurysms (35). In the subsequent hemodynamic and morphological studies using mirror aneurysm models, all reported that higher LSA and lower WSS were associated with aneurysm rupture with or without bifurcations (19, 29). Low WSS could degenerate endothelial cells by triggering atherosclerotic and inflammatory pathways, ultimately causing IA rupture. These findings agree with those of the present study.

One of the issues concerning the ruptured mirror PComAAs was that the morphological parameters might be affected by sentinel spasm. Changes in morphology parameters could lead to changes in hemodynamic parameters. Vasospasm, which influenced vascular morphology (parent artery), usually occurs on the fifth day of SAH (36). All the ruptured mirror PcomAAs were diagnosed using DSA within 48 h of subarachnoid SAH. Accordingly, vasospasm rarely affected the calculation of morphological and hemodynamic parameters.

There are some potential limitations in our study. First, it was a retrospective study. Although we compared ruptured aneurysms with unruptured aneurysms using ruptured mirror PComAAs, we, nevertheless, ignored that the ruptured event influences the results. Second, whereas our study used the most significant number of ruptured mirror PComAAs, all the patients with mirror ruptured PComAAs came from a single center, and the 72 ruptured mirror PComAAs were still a relatively small number. In the future study, we look forward to multicenter studies with larger sample sizes to validate our result. Third, to establish the patient-specific ruptured and unruptured aneurysm models, some small vessel branches far away from the aneurysms were artificially moved. Fourth, while patient-specific models were applied in the CFD simulation, the inlet boundary conditions were not patient specific, and the assumptions of laminar flow, Newtonian blood flow, and rigid wall were used in our hemodynamic simulations. Doing so may lead to inaccurate results and conclusions. Fifth, although our study obtained some meaningful color images, hemodynamic parameters using different thresholds and different grid drawing methods may generate various figures. These cause the meaning of these colorful maps to become uncertain. Finally, due to the complexity and time-consuming process of current CFD studies and the omission of several variables in the equation, its application in clinical work is limited. CFD simulation needs to be simplified, and some equations need to be developed to measure multiple variables in future studies to allow decision making in a timely fashion.

## Conclusion

We compared eight hemodynamical and 14 morphological parameters between ruptured and unruptured aneurysms with 72 ruptured mirror PComAAs. SR was the first most significant parameter associated with IA rupture, whereas AR, NWSS, mean WSS, and LSA were independent parameters characterizing the status of IA rupture. These findings may facilitate the management of the unruptured aneurysms. We hope that these results will provide data to guide future studies and appropriate treatment strategies.

## Data Availability Statement

The original contributions presented in the study are included in the article/supplementary material, further inquiries can be directed to the corresponding authors.

## Ethics Statement

Written informed consent was obtained from the individual(s) for the publication of any potentially identifiable images or data included in this article.

## Author Contributions

JY, XF, and CH contributed to the conception, design, and drafted the manuscript. XJ, ZL, and XZ collected and analyzed the data. DW, NL, DX, and JL made critical revisions to the manuscript. BZ, DX, and FQ contributed to the final approval of the version to be published. All authors contributed to the article and approved the submitted version.

## Funding

This work was supported by grants from Young and Middle-Aged Natural Science Foundation of Wannan Medical College (No. WK2021F26), Domestic Visiting Scholar Program for Excellent Young Talents in Colleges and Universities of Anhui Province (No. gxgnfx2021125), the Science Research Project of Professional of the First Affiliated Hospital of Wannan Medical College (No. YR201911), Anhui Provincial Natural Science Foundation (No. 2008085QH421), and the key Scientific Research Project of Wannan Medical College (No. WK2019ZF02).

## Conflict of Interest

The authors declare that the research was conducted in the absence of any commercial or financial relationships that could be construed as a potential conflict of interest.

## Publisher's Note

All claims expressed in this article are solely those of the authors and do not necessarily represent those of their affiliated organizations, or those of the publisher, the editors and the reviewers. Any product that may be evaluated in this article, or claim that may be made by its manufacturer, is not guaranteed or endorsed by the publisher.
